# Dobutamine “Stress” Test and Latent Cardiac Susceptibility to Inhaled Diesel Exhaust in Normal and Hypertensive Rats

**DOI:** 10.1289/ehp.1104684

**Published:** 2012-04-27

**Authors:** Mehdi S. Hazari, Justin Callaway, Darrell W. Winsett, Christina Lamb, Najwa Haykal-Coates, Q. Todd Krantz, Charly King, Daniel L. Costa, Aimen K. Farraj

**Affiliations:** 1 Environmental Public Health Division, National Health and Environmental Effects Research Laboratory, U.S. Environmental Protection Agency, Research Triangle Park, North Carolina, USA; 2 Curriculum in Toxicology, University of North Carolina–Chapel Hill, Chapel Hill, North Carolina USA; 3 National Health and Environmental Effects Research Laboratory, Office of Research and Development, U.S. Environmental Protection Agency, Research Triangle Park, North Carolina, USA

**Keywords:** air pollution, arrhythmia, cardiac, diesel exhaust, dobutamine, “stress” test

## Abstract

Background: Exercise “stress” testing is a screening tool used to determine the amount of stress for which the heart can compensate before developing abnormal rhythm or ischemia, particularly in susceptible persons. Although this approach has been used to assess risk in humans exposed to air pollution, it has never been applied to rodent studies.

Objective: We hypothesized that a single exposure to diesel exhaust (DE) would increase the risk of adverse cardiac events such as arrhythmia and myocardial ischemia in rats undergoing a dobutamine challenge test, which can be used to mimic exercise-like stress.

Methods: Wistar-Kyoto normotensive (WKY) and spontaneously hypertensive (SH) rats implanted with radiotelemeters and a chronic intravenous catheter were whole-body exposed to 150 μg/m^3^ DE for 4 hr. Increasing doses of dobutamine, a β_1_-adrenergic agonist, were administered to conscious unrestrained rats 24 hr later to elicit the cardiac response observed during exercise while heart rate (HR) and electrocardiogram (ECG) were monitored.

Results: A single exposure to DE potentiated the HR response of WKY and SH rats during dobutamine challenge and prevented HR recovery at rest. During peak challenge, DE-exposed SH rats had lower overall HR variability when compared with controls, in addition to transient ST depression. All DE-exposed animals also had increased arrhythmias.

Conclusions: These results are the first evidence that rats exhibit stress-induced cardiac dysrhythmia and ischemia sensitivity comparable to humans after a single exposure to a toxic air pollutant, particularly when in the presence of underlying cardiovascular disease. Thus, exposure to low concentrations of air pollution can impair the heart’s ability to respond to stress and increase the risk of subsequent triggered dysfunction.

Numerous epidemiological studies have demonstrated that chronic exposure to air pollution increases cardiovascular morbidity and mortality, but also that acute exposure triggers adverse cardiac events such as arrhythmia or infarction (Dockery et al. 1993; Hoek et al. 2001). Studies examining the acute effects of transient exposure have identified persons with cardiovascular conditions such as ischemic heart disease, heart failure, or hypertension as being particularly susceptible to ambient air pollution (Goldberg et al. 2001; Hoek et al. 2001) frequently linked to vehicular traffic. Of the vehicular emissions, diesel exhaust (DE) has been found to be particularly noxious (Zielinska et al. 2004) because of its disproportionate richness in toxic compounds.

Despite the high toxicity of various air pollution mixtures, direct symptoms are not always apparent, belying the full risk of exposure. As such, the degree to which air pollution may sensitize or prime the host to a subsequent chemical or physical challenge has not been widely considered among the risks of air pollution. In the case of cardiovascular risk associated with air pollution, the impact is often an event and frequently occurs with some latency period. Hence, the relationship of the event with air pollution may be missed or otherwise not appropriately appreciated. Indeed, this latent effect may reflect increased cardiovascular risk associated with the air pollution exposure, but its assessment cannot occur without first recognizing that a trigger may be needed to manifest a response.

Exercise “stress” testing, which is performed to determine the amount of stress that a patient’s heart can tolerate before developing adverse responses, has been used for many years in clinical settings to evaluate suspected or known heart disease. Dobutamine—a direct-acting, sympathomimetic drug that stimulates β_1_-adrenergic receptors on the heart, resulting in an increase in heart rate (HR) and contractility—can be administered intravenously (iv) as a cardiac stress test in place of exercise, especially in patients with advanced cardiac disease who may not be capable of physical exercise. Regardless of the method, the normal response to an exercise stress test is measured as elevations in HR and blood pressure (BP) that are proportional to the increased exertion, which also then reflects in the expected recovery at cessation. Results suggestive of potential cardiac dysfunction, including HR abnormalities or electrical problems such as arrhythmias, are those that reveal excessive stress on the heart during exercise. Although largely used in patients complaining of chest pain or other symptoms characteristic of heart disease, research has shown that exercise stress testing can be used to evaluate susceptibility in asymptomatic persons who display insidious risk due to factors such as smoking or high cholesterol (Gibbons et al. 1997). The evidence is convincing that air pollution is a factor that also increases the risk of cardiovascular morbidity and mortality (Brook et al. 2010). For instance, Lanki et al. (2006) showed that combustion-derived, traffic particulate matter (PM) caused greater ST depression, a putative indicator of myocardial ischemia, in patients with ischemic heart disease when compared with untreated healthy controls. Mills et al. (2007) reported observing similar results with DE in men with coronary artery disease. Both studies superimposed exercise as a stressor to trigger the ST depression phenomenon.

Although these and many other studies have documented the deleterious cardiopulmonary effects of exposure to air pollution during exercise (which could perhaps be due to increased dose with increased ventilation), work is needed to ascertain whether adverse responses may be triggered by subsequent exercise stress in the days after a single or short-term exposure. This is particularly important given the uncertainties surrounding the time-lag of cardiovascular effects identified by several epidemiological studies and the decreased perception of risk that might occur with the end of a high air pollution event. Thus, we have adapted a stress test regimen in conscious and unrestrained rats that might serve as a tool to evaluate cardiovascular risk and aid in understanding the full nature of air pollution health effects. Using the dobutamine challenge test, the objective of this study was to simulate the cardiac stress of exercise to evaluate its utility as a trigger of adverse responses and unmask the latent effects of a complex multipollutant atmosphere such as DE using healthy and cardiac-compromised rat models. In contrast to healthy animals, we hypothesized that rats with underlying cardiovascular disease would show prominent signs of cardiac dysfunction during a dobutamine challenge and that a single exposure to DE would potentiate the adverse response.

## Methods

*Animals.* In the study we used 12-week-old male Wistar-Kyoto (WKY) and spontaneously hypertensive (SH) rats (Charles River, Wilmington, MA) weighing 300–400 g. Upon arrival, animals were housed two per cage on woodchip bedding in a facility approved by the Association for Assessment and Accreditation of Laboratory Animal Care International (AAALAC). Animals were housed singly after surgery until exposure as well as between the end of exposure and experimentation. All animals were provided food and water *ad libitum* at all times except during exposure. All animals were treated humanely and with regard to alleviation of suffering. All experimental protocols were approved by and in accordance with the guidelines of the Institutional Animal Care and Use Committee of the U.S. Environmental Protection Agency.

*Experimental groups.* Rats were assigned to one of the following groups: *a*) WKY exposed to filtered air (FA); *b*) WKY exposed to 150 µg/m^3^ DE; *c*) SH exposed to FA; and *d*) SH exposed to 150 µg/m^3^ DE. For each group, *n* = 5 or 6.

*Radiotelemetry.* Radiotelemeters were implanted using aseptic surgical technique as previously described (Hazari et al. 2009). Briefly, animals were weighed and anesthetized with ketamine hydrochloride/xylazine hydrochloride solution (1 mL/kg, intraperitoneally; Sigma-Aldrich, St. Louis, MO). Each animal was implanted with a radiotelemetry transmitter (TA11CTA-F40; Data Sciences International, St. Paul, MN) in the abdominal cavity with the electrode leads guided and secured in positions approximating lead II of a standard electrocardiogram (ECG). [For more detail, see Supplemental Material, pp. 3–4 (http://dx.doi.org/10.1289/ehp.1104684)].

*Implantation of iv catheter.* Immediately after implantation of the radiotelemeter and closure of the abdominal muscle and skin, and while still anesthetized, animals were surgically implanted with an iv catheter into the left jugular vein. Catheters were filled with a heparin/saline solution from the time of implantation until experimentation and were flushed periodically (Heiser 2007). [See Supplemental Material, pp. 4–5 (http://dx.doi.org/10.1289/ehp.1104684)].

*DE generation and exposure.* The method for DE generation has been previously described [Hazari et al. 2011; see also Supplemental Material, pp. 5–6 (http://dx.doi.org/10.1289/ehp.1104684)]. Briefly, DE for exposure experiments was generated with a diesel generator (Yanmar Co. Ltd., Osaka, Japan) using low-sulfur diesel fuel (32 ppm). Target DE concentration, which was based on the fine PM (PM_2.5_; mass median aerodynamic diameter < 2.5 µm) fractions of the diluted exhaust, was 150 μg PM/m^3^; this was routed to an unfiltered exposure chamber. Control animals were placed in a third chamber supplied with HEPA-filtered room air (FA). Continuous emission monitors (CEMs) measured chamber concentrations of PM, oxygen (O_2_), carbon monoxide (CO), nitrogen oxides, and sulfur dioxide (SO_2_) [exposure details were similar to what has been previously reported (Hazari et al. 2011)]. Animals were exposed for 4 hr.

*Dobutamine challenge test.* Experimentation was conducted 24 hr after exposure; the heparinized saline was withdrawn from the iv catheter and replaced with saline only. Ventilatory function was measured in a whole-body plethysmograph (WBP; model PLY3213, Buxco Electronics, Inc., Wilmington, NC); all animals were previously acclimated to the plethysmograph 2 days before exposure. HR and ECG were measured and recorded continuously from the radiotelemeter. The iv catheter was exteriorized from the WBP and connected to an infusion pump for the delivery of dobutamine. After the animals became settled (which took approximately 5–10 min), a 5-min baseline period was recorded, and then increasing doses of dobutamine (20, 40, 80, 160, and 320 µg/kg; Sigma-Aldrich) were infused (0.2 mL/min for 2 min). A 2-min recovery period was used between dobutamine doses. Thus, the animals were monitored for increases in HR, changes in ECG (particularly occurrence of arrhythmias), and then responses during post-challenge recovery.

*Ventilatory function.* The WBP continuously and noninvasively monitored ventilatory function in all animals during the dobutamine challenge test. The plethysmograph pressure was monitored using Biosystems XA software (Buxco Electronics, Inc.). Using respiratory-induced fluctuations in ambient pressure, breathing frequency (*f*) was calculated and recorded on a breath-by-breath basis and averaged over 10-sec intervals (Buxco Electronics, Inc. 2005).

*Radiotelemetry data acquisition and ECG analysis.* Radiotelemetry methodology was used in this study to track changes in cardiovascular function by monitoring ECG, HR, and core body temperature (Tco). This methodology provided continuous monitoring and collection of physiologic data from unrestrained, unanesthetized rats. Data signals were transmitted from surgically implanted radiotelemeters to a remote receiver (DataART2.1; Data Sciences International, Inc.) located under the WBP. HR was obtained from the ECG waveform. ECGAuto software (emka technologies, Inc., Falls Church, VA) was used to visualize individual ECG signals and analyze and measure ECG segment durations and amplitudes. Heart rate variability (HRV) was also calculated in the time-domain; the standard deviation of the normal-to-normal intervals (SDNN) provides a measure of total variability and the square root of the mean squared of successive NNs (RMSSD) quantifies the standard deviation of differences between neighboring intervals. Frequency-domain measures were also determined using the fast Fourier transform algorithm, including low frequency (LF; 0.200–0.750 Hz), high frequency (HF; 0.750–2.00 Hz), and the ratio of these two frequency-domains (LF/HF). Lastly, cardiac arrhythmias were identified qualitatively based on our previous findings (Farraj et al. 2011) and the Lambeth convention criteria (Walker et al. 1988), counted, and totaled for the duration of the dobutamine challenge (baseline, treatment, and recovery). [See Supplemental Material. Figure S1 (http://dx.doi.org/10.1289/ehp.1104684) for examples of arrhythmias typically found in the animals in this study.]

*Statistics.* The statistical analyses for all data in this study were performed using SAS version 9.1.3 software (SAS Institute Inc., Cary, NC). PROC MIXED and PROC GLIMMIX procedures were used to analyze all ECG- and HRV-generated data. Tests of normality were performed for all continuous variables, and parametric methods of analysis were used. A linear mixed model with restricted maximum-likelihood estimation analysis (SAS Institute Inc.) and the least squares means post hoc test were used to determine statistical differences for all data. All dobutamine dose–response data were analyzed using an analysis of variance (ANOVA) for repeated measures. *p* < 0.05 was considered as statistically significant. Reported values represent mean ± SE.

## Results

*Core body temperature.* Tco was lower at baseline in rats exposed to DE when compared with FA. All rat groups (FA and DE) experienced an increase of ≥ 0.75 to 1.0°C in Tco during the dobutamine challenge test [see Supplemental Material, Figure S2 (http://dx.doi.org/10.1289/ehp.1104684)].

**Figure 1:**
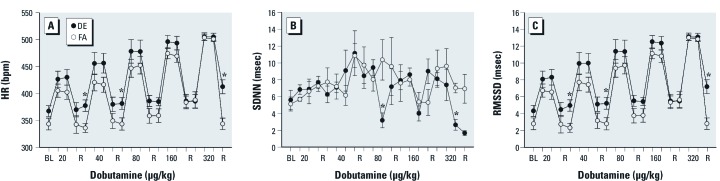
A single exposure to DE prevents HR recovery and causes decreased overall HRV in WKY rats during dobutamine challenge. Abbreviations: BL, baseline; bpm, beats per minute; R, reference. Values are presented as mean ± SE. Dobutamine dose-dependently increased HR in conscious/unrestrained WKY rats exposed to FA; this response was not significantly different in WKY rats exposed to DE; however, recovery HR was significantly higher in WKY exposed to DE when compared with those exposed to FA (FA controls) (*A*). At the highest dobutamine dose, the decrease in SDNN (*B*) was greater in DE-exposed WKY rats when compared with FA controls. RMSSD also decreased significantly at the highest dose (*C*). * *p* < 0.05 compared with FA controls; *n* = 5–6.

**Figure 2:**
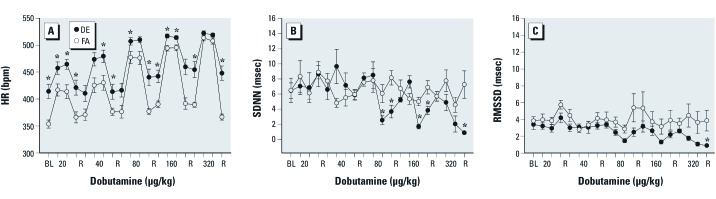
A single exposure to DE increases the peak HR response, decreases overall HRV, and prevents HR recovery in SH rats during dobutamine challenge. Dobutamine dose-dependently increased HR in conscious/unrestrained SH rats exposed to FA (*A*). Although the relative increase in HR at each dose of dobutamine was similar between FA- and DE-exposed animals, the peak HR at each dose (except 320 µg/kg) and at recovery were significantly increased in SH rats exposed to DE when compared with those exposed to FA (FA controls) (*A*). At the high dobutamine doses, the decrease in SDNN (*B*) was significantly greater in DE-exposed rats when compared with those exposed to FA. RMSSD was only significantly decreased in DE-exposed SH rats during the final recovery (*C*). Values are presented as mean ± SE. *p < 0.05 compared with FA controls; *n* = 5–6.

*Heart rate.* We observed no difference in the baseline (pre-challenge) HR of WKY rats exposed to DE when compared with those exposed to FA (Figure 1A); whereas the baseline HR of DE-exposed SH rats was significantly higher than the HR of control rats (Figure 2A). In FA-exposed WKY and SH rats, dobutamine infusion caused a dose-dependent increase in HR, which returned to near baseline levels during recovery. The HR response of WKY rats exposed to DE did not differ from controls; however, the HR was significantly increased during post-challenge recovery (Figure 1A). DE-exposed SH rats had significantly increased HR during challenge and post-challenge recovery (Figure 2A), but there was no statistically significant difference between strains. The HR of both DE-exposed strains remained elevated during post-challenge recovery when compared with their baseline values.

*Heart rate variability.* In WKY rats, DE exposure caused a decrease in the SDNN at the 80- and 320-µg/kg doses of dobutamine when compared with FA (Figure 1B), whereas RMSSD was only decreased significantly at the highest dose (Figure 1C). In SH rats exposed to DE, SDNN (Figure 2B) decreased significantly during the 80-, 160-, and 320-µg/kg doses of dobutamine when compared with controls and RMSSD also decreased, though not significantly (Figure 2C). The frequency-domain HRV parameters did not change significantly (not shown) nor was there any significant difference between the strains in any endpoint.

**Figure 3:**
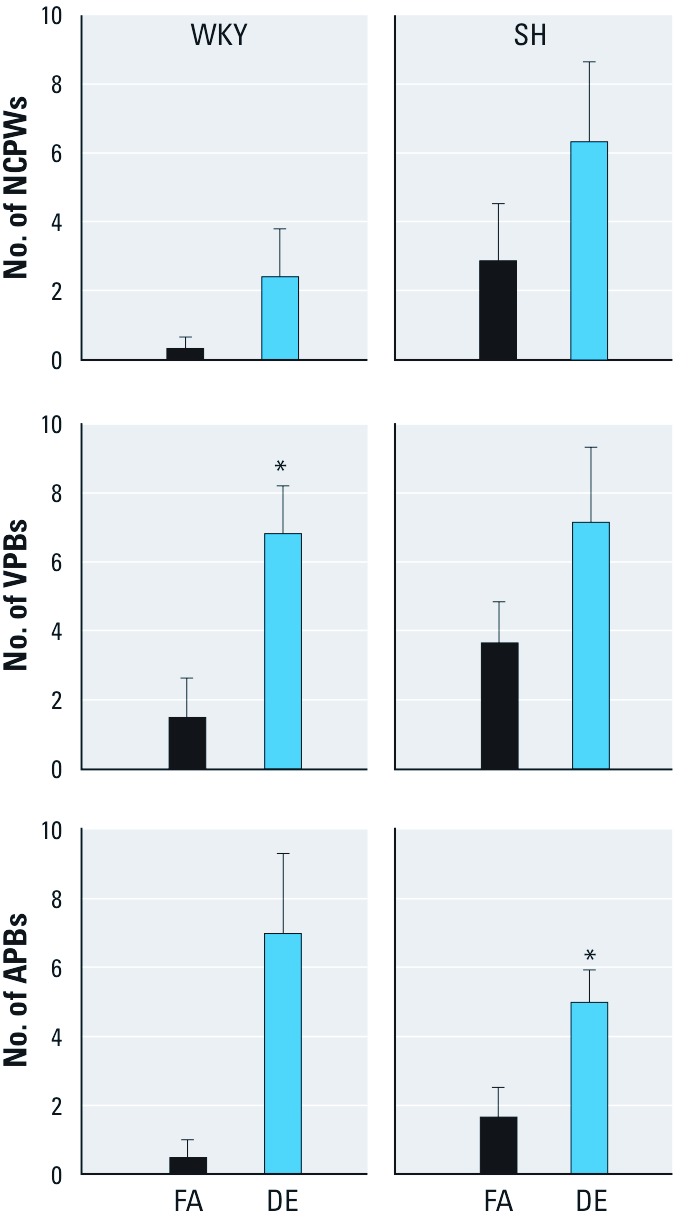
A single exposure to DE increases the number of cardiac arrhythmias in rats during dobutamine challenge. Abbreviations: APBs, atrial premature beats; NCPWs, non-conducted p-waves; VPBs, ventricular premature beats. Values are presented as mean ± SE. Exposure to DE significantly increased the number of VPBs in WKY rats and APBs (atrial premature beats) in SH rats during dobutamine challenge when compared with those exposed to FA (controls). *p < 0.05 compared with FA controls; *n* = 5–6.

*Frequency of arrhythmia.* WKY rats exposed to DE had a greater number of VPBs (ventricular premature beats) than FA controls, whereas SH rats exposed to DE had a significantly greater number of APBs (atrial premature beats) (Figure 3). There was no difference in arrhythmia counts between the strains.

*Breathing frequency.* Infusion of dobutamine caused *f* to increase in all rats. Baseline *f* was significantly higher in WKY and SH rats exposed to DE when compared with FA; however, the response during the challenge and after were not different among the groups [see Supplemental Material, Figure S3A,B (http://dx.doi.org/10.1289/ehp.1104684)].

*Electrocardiogram.* Supplemental Material, Figure S4 (http://dx.doi.org/10.1289/ehp.1104684) shows the changes in ECG during dobutamine challenge. There was no difference in the PR interval of WKY and SH rats, whether exposed to FA or DE. Although not statistically significant, QRS intervals were generally longer in SH rats when compared with WKY rats. Exposure to DE did not significantly change any ECG parameter in either strain.

**Figure 4:**
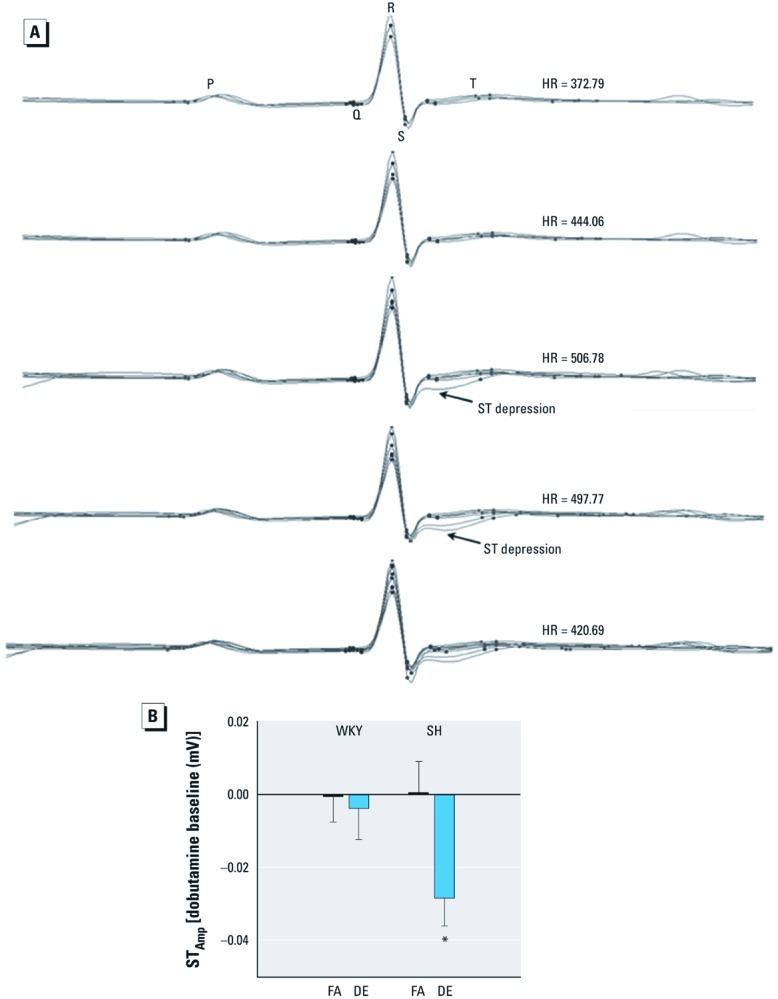
SH rats develop ST depression at peak HRs during dobutamine challenge test. (*A*) Sample ECG waveforms (overlaid) from one of the DE-exposed SH rats show the development of ST depression as HR peaks at the highest dose of dobutamine; circles represent ECG segments (P wave, Q wave, R wave, S wave, T wave). (*B*) Changes in ST amplitude from baseline for WKY and SH rats exposed to FA and DE at peak HR during the highest dose of dobutamine in the challenge test. Values are presented as mean ± SE. *p < 0.05 compared with FA controls; *n* = 5–6.

All SH rats exposed to DE experienced ST depression at peak HR during the highest dobutamine dose (Figure 4A shows ST depression in a representative DE-exposed SH rat). ST depression at peak HR was measured as a decrease in the ST amplitude from baseline and was statistically significant for DE-exposed SH, not WKY, rats. (Figure 4B). In comparison, only 1 FA-exposed SH rat, 3 DE-exposed WKY rats, and 1 FA-exposed WKY rat had any evidence of ST depression (data not shown).

## Discussion

These experiments show that a single exposure to DE causes cardiac dysfunction in both normotensive WKY and spontaneously hypertensive SH rats during dobutamine challenge. Even though both strains had adverse responses, SH rats still appeared to be more susceptible to cardiac stress than the healthy animals as indicated by the higher postexposure HRs and ST depression. Therefore, just as in humans, challenge or stress testing can be a valuable tool in assessing the potential risk of adverse cardiac events in rats after air pollutant exposure, particularly when overt symptoms of exposure are not observed. Moreover, this challenge model can be used to investigate the fundamental biology of this cardiac sensitivity to better understand the human response scenario and allow researchers to better measure the effects of lower air pollution concentrations.

Not only is exercise stress testing used to identify and assess persons with existing or suspected cardiovascular disease, but also persons who have risk factors associated with sudden development of disease. Tofler and Muller (2006) suggested that in such a case, a trigger, which is an “activity that produces short-term physiological change, such as a surge in arterial pressure or heart rate,” can set off adverse cardiac effects. Thus, DE, like any other air pollutant, might “set the stage” for a future response. As such, the utility of stress or challenge testing is in its ability to act as a trigger and reveal the very subtle changes caused by the air pollutant, e.g., changes in the capacity to compensate for stress.

The lack of HR recovery in our rats might represent such a subtle change. The lack of HR recovery was not entirely surprising because HR profiles during exercise and recovery are considered a predictor of sudden adverse cardiac events in humans. Over a 23-year follow-up period, researchers found that the risk of suddenly dying from myocardial infarction was significantly increased in subjects with higher resting HR and especially in those with impaired decrease of HR after the termination of exercise (Jouven et al. 2005). Thus the symptoms associated with heightened risk observed in DE-exposed rats during dobutamine challenge parallels human responses to exercise stress testing. As might be the case in our rats, Jouven et al. (2005) suggested autonomic imbalance and reflex sympathetic activation as contributors to these deleterious effects. Regardless, these responses in humans and rats are indicative of a decreased capacity to respond to stress and, hence, a decreased capacity to compensate within normal homeostatic bounds. In humans, repolarization changes, which also occur in rats (Kapur et al. 2010), are often seen during exercise and can predispose a person to arrhythmias in the presence of cardiovascular disease (Korantzopoulos et al. 2011).

The fact that DE changed the HR recovery profile during the dobutamine challenge of both WKY and SH rats indicates that even a single exposure might begin to contribute to increased cardiac risk. In a study of healthy traffic police officers exposed to urban pollutants, researchers found that post-exercise recovery of cardiopulmonary function, including HR and BP, was significantly better in control subjects than in subjects in the exposed group (Volpino et al. 2004). In this study, there were some differences in the dobutamine responses of the SH rats when compared with WKY (e.g., higher peak HR, decreased recovery); however we could not draw any conclusions about the strains’ HR responsiveness (i.e., amount of increase) to dobutamine because even though the peak responses of SH rats were higher than WKY rats, the baseline HR of SH rats was already elevated. Although it was not measured, we assume both WKY and SH rats had an increase in BP during dobutamine challenge because dobutamine has been shown to cause an increase in BP in normal Wistar rats (Plante et al. 2005) and also, in fact, the same occurs in healthy young humans (Velez-Roa et al. 2003). However, SH rats have greater increases in BP in response to adrenalin (Yamaguchi and Kopin 1980) and proportional increases in BP in response to isoprenaline (Weinstock and McCarthy 1983) compared with WKY rats and hence might have had a higher peak BP response than WKY rats during dobutamine administration despite the preexisting hypertension.

Higher BP during the challenge may explain the response of the SH strain. Dobutamine is known to increase HR and BP artificially and also to blunt baroreceptor function (van de Borne et al. 1999; Zaza and Lombardi 2001), which normally would elicit a vagal reflex when BP increases. Van de Borne et al. (1999) found that the largest decreases in baroreflex sensitivity (BRS) occurred during the largest increases in BP induced by dobutamine. Thus, greater increases in BP during dobutamine administration and preexisting reduced BRS (Struyker-Boudier et al. 1982) may explain the upward shift in baseline and peak HR of the SH rats when compared with the WKY strain. Consequently, exposure to DE, which has been shown to increase vasoconstriction (Peretz et al. 2008), might have further increased the effect in both strains in these experiments. Similar results have been seen with lead, which has also increased BP in rats and caused higher HR responses to dobutamine and isoproterenol than seen in unexposed rats (Reza et al. 2009).

Air pollution is also associated with a decrease in HRV (Devlin et al. 2003; Pope et al. 1999), which according to the Framingham Heart Study, is associated with increased cardiac risk (Tsuji et al. 1996). Although not measured immediately postexposure, HRV was significantly reduced in DE-exposed rats during the dobutamine challenge. Paradoxically, there was no difference in SDNN between WKY groups at the second highest dose of dobutamine, not because there was no decrease in the DE group, but rather because there was also a decrease in the FA group SDNN at this dose, which could have been due to an increase (i.e., a reset) in their between-dose resting HR. However, given there were no significant changes in LF/HF during the dobutamine challenge, it is difficult to rule out that the observed changes in SDNN were due to the direct effect of the drug on sinus node pacemaker activity. In fact, mean HR is one of the two strongest determinants of SDNN (Tsuji et al. 1996). Thus, the direct effects of dobutamine on HR might confound the response of LF/HF to the influences of neural activity (Zaza and Lombardi 2001) and mask changes in autonomic activity. Instead, a change identified as “autonomic” may have been more justified if LF/HF was also affected by dobutamine. Similarly, a limitation of this study is that we used an HF range to 2 Hz rather than 3.5 Hz [or even the Nyquist (maximum) frequency], which might have better captured changes in HF, particularly given the increased *f* during dobutamine exposure. Future studies will need to account for these factors to better elucidate HRV effects. However, it is possible that the HRV effect observed in our rats represents an alteration of cellular response to autonomic agonists rather than a change in autonomic neural activity. Regardless, the DE-induced potentiated effects at the higher doses of dobutamine reflect a shift from the normal function of the heart. Therefore, the results obtained during challenge might be relevant for periods of stress given no HRV changes were observed during or after exposure to DE in the same strains of rat (Lamb et al. 2012) and because time-domain HRV has been shown to predict cardiovascular risk irrespective of the pathophysiological interpretation.

Sudden decreases in HRV have been observed prior to fatal ischemic events, which are often indicated by ST depression on the ECG (Pozzati et al. 1996). As far as air pollution effects in exercising humans are concerned, even short-term exposures increase the risk of myocardial ischemia and observable ST depression (Lanki et al. 2008; Mills et al. 2007). This is the first study to demonstrate similar worsening of ST depression in the ECG of air pollution-exposed rats undergoing stressful challenge. The brief nature of our challenge regimen and the young age of our animals may explain why decreased ST amplitude, a quantitative measure of ST depression, only appeared at the highest dose of dobutamine. Nevertheless, the fact that all SH rats, but no WKY rats, exposed to DE experienced qualitative, albeit transient, ST depression during peak HR reflects not only the susceptibility of this hypertensive strain for cardiac dysfunction but also the ability of DE to increase the risk of myocardial ischemia.

Lanki et al. (2008) assessed the time trends of effects in their subjects, and although they explained the effect of air pollution on ST depression at 2-day lag by increased pulmonary and resulting systemic inflammation, we did not see similar inflammation in our rats at 24 hr postexposure (Lamb et al. 2012). Although not measured, there is the possibility that 1 day after exposure, reactive oxygen species mediate changes in autonomic reflexes and lead to impaired endothelial function, vasoconstriction, and decreased oxygen supply; in fact, this has been shown to be a mechanism of particle effects (Verrier et al. 2002). Other than ST depression, ECG results were generally unremarkable and not statistically significant. However, the trend of longer QRS durations in the SH rats, as compared with WKY, and prolonged QTc in SH rats after DE exposure might explain the heightened arrhythmia risk [reviewed by Farraj et al. (2011)] and indicate a greater chance for ventricular repolarization abnormalities, which indeed have been shown in other air pollution studies (Liao et al. 2010).

Overall, the resulting adverse responses (i.e., arrhythmogenesis, HR changes, ST depression) appear to be similar to those previously observed in our laboratory with other pollutants, suggesting the effects of inhaled toxicants on the heart may be nonspecific. In humans, increases in CO, nitrogen dioxide (NO_2_), or PM_10_ have been found to be associated with a same-day increase in arrhythmia (Peters et al. 2000). In fact, a wide variety of air pollutants entering into the airways can alter neural reflexes, either through particulate irritation or through gaseous chemical activation of several different nerve endings, leading to autonomic imbalance and myocardial electrical disruption thereafter. Other possible contributors include an increase in BP (Brook and Rajagopalan 2009), which was observed 24 hr after rats were exposed to DE in a separate study in our laboratory, and increased oxidative stress and inflammation (reviewed by Brook 2008; Simkhovich et al. 2008).

## Conclusions

The data presented here show that a single exposure to a complex multipollutant primes the host to subsequent triggered cardiovascular responses. Our findings of increased arrhythmogenesis and decreased HR recovery during challenge confirm that air pollution may well condition persons—especially those with underlying disease—lowering their response thresholds to otherwise typical triggers that would be compensated within normal homeostatic bounds. This lack of compensatory capacity is indicated by an altered stress responsiveness of the heart, which may precipitate from a number of causes such as airway sensory nerve reflex activation and/or the systemic stress of exposure. These results also point to the utility of challenge testing, particularly exercise stress testing, which clinical researchers have used to measure the risk of air pollution conditioning or altered threshold. Given the similarity of results observed in our rats to those obtained in humans, we suggest here that rodent challenge testing may facilitate extrapolation of rodent data to humans and thereby aid in the assessment of multipollutants and confirm causality for specific components.

## Supplemental Material

(463 KB) PDF
