# Supplementary material for: Dobutamine “Stress” Test and Latent Cardiac Susceptibility to Inhaled Diesel Exhaust in Normal and Hypertensive Rats
Source: Environ Health Perspect. 2012 Apr 27;120(8):1088–93. doi: 10.1289/ehp.1104684 (PMC3440084; doi:10.1289/ehp.1104684)
Supplement: (463 KB) PDF [file ehp.1104684.s001.pdf]

SUPPLEMENTAL MATERIAL

Dobutamine "Stress" Test and Latent Cardiac Susceptibility to Inhaled Diesel Exhaust in Normal and Hypertensive rats

Mehdi S. Hazari[1], Justin Callaway[3], Darrell W. Winsett[1], Christina Lamb[3], Najwa Haykal-Coates[2], Q. Todd Krantz[1], Charly King[1], Daniel L. Costa[2] and Aimen K. Farraj[1]

[1]Environmental Public Health Division, [2]Office of Research and Development, United States Environmental Protection Agency, Research Triangle Park, NC, 27711; [3]University of North Carolina, Chapel Hill, NC 27599

Disclaimer: This paper has been reviewed and approved for release by the National Health and Environmental Effects Research Laboratory, U.S. Environmental Protection Agency. Approval does not signify that the contents necessarily reflect the views and policies of the U.S. EPA, nor does mention of trade names.

Corresponding author: Mehdi S. Hazari, Environmental Public Health Division, USEPA, 109 Alexander Drive, B105; Research Triangle Park, NC 27711; (Phone: 919-541-4588; Fax: 919-541-0034; email: hazari.mehdi@epa.gov)

Running title: Stress test reveals latent effects of diesel exhaust

TABLE OF CONTENTS

I. Materials and methods

A. Full description of *Radiotelemetry*

B. Full description of *Intravenous Catheter Implantation*

C. Full description of *Diesel Exhaust Generation and Exposure*

II. Figures

A. Supplemental Material, Figure S1. Typical electrocardiogram (ECG) traces showing rat cardiac arrhythmias.
B. Supplemental Material, Figure S2. Post-exposure core body temperature response during dobutamine challenge
C. Supplemental Material, Figure S3. Exposure to diesel exhaust increases the baseline breathing frequency of WKY and SH rats.
D. Supplemental Material, Figure S4. Electrocardiographic changes during dobutamine challenge

III. References

I. Materials and methods

A. *Radiotelemetry*. Radiotelemeters were implanted in all animals as previously described (Hazari et al. 2009); this methodology was used to track changes in cardiovascular function by monitoring ECG and HR. Briefly, animals were weighed and anesthetized with ketamine hydrochloride/xylazine hydrochloride solution (1ml/kg, ip Sigma-Aldrich, St. Louis, MO). Using aseptic technique, each animal was implanted with a radiotelemetry transmitter (Model TA11CTA-F40, Data Sciences International, St. Paul, MN) in the abdominal cavity through a small incision. The electrode leads were guided through the abdominal musculature via separate stab wounds and tunneled subcutaneously across the lateral ventral thorax; the distal portions of the leads were secured in positions that approximated those of the lead II of a standard electrocardiogram (ECG). Body heat was maintained both during and immediately following the surgery. All animals were allowed 7-10 days to recover from the surgery and reestablish circadian rhythms. Using a remote receiver (DataART2.1: Data Sciences International, Inc., St. Paul, MN), ECG waveforms could be continuously acquired and saved during the 5-min; HR was obtained from the ECG.

ECGAuto software (EMKA technologies USA, Falls Church, VA) was used to visualize individual ECG signals, analyze and quantify ECG segment durations and identify cardiac arrhythmias. Using ECGAuto, P wave, QRS complex, and T wave were identified for individual ECG waveforms and compiled into a library and used for analysis of all experimental ECG traces. The following parameters were determined for each ECG waveform: PR interval, QRS duration, QT corrected for HR (QTc) using Bazett's formula, corrected ST amplitude and ST interval. The Lambeth conventions (Walker et al. 1988) were used as guidelines for the identification of cardiac arrhythmic events in rats. Arrhythmias were identified as either atrial premature beats (APBs), ventricular

premature beats (VPBs), non-conducted P-waves (NCPW), or ventricular tachycardia (VT). The number of arrhythmias that occurred during the dobutamine challenge (including baseline and recovery) were counted for each animal.

Heart rate variability (HRV) was also calculated as the mean of the differences between sequential HRs for the complete set of ECG signals. For each 1-min stream of ECG waveforms, mean time between successive QRS complex peaks (RR interval), mean HR, and mean HRV-analysis–generated time-domain measures were acquired. The time-domain measures included standard deviation of the time between normal-to-normal beats (SDNN), and root mean squared successive differences (RMSSD). HRV analysis was also conducted in the frequency domain using a fast-Fourier transform. In this study, the spectrum was divided into low-frequency (LF) and high-frequency (HF) regions. The ratio of these two frequency domains (LF/HF) was calculated as an estimate of the relative balance between sympathetic (LF) and vagal (HF) activity.

B. *Implantation of Intravenous Catheter.* Immediately after implantation of the radiotelemeter and closure of the abdominal muscle and skin, while still anesthetized, animals were surgically implanted with an intravenous catheter. After assurance that proper anesthetic plane was still maintained (toe pinch), the neck was shaved, wiped down with alcohol swabs and draped. The left jugular vein was exposed and isolated using blunt, curved forceps, and a heparin-filled (50 U/ml of saline) catheter (PE50) was inserted into the left jugular vein for administration of drugs. The tip of the catheter was advanced until close to the right atrium and anchored and secured to the muscle tissue using surgical suture. The catheters were tunneled subcutaneously, exteriorized at the back of the neck, and sealed with stainless steel pins. Catheters were filled with the heparin/saline solution from the time of implantation until experimentation and flushed periodically. The incision was

closed with sutures and surgical staples, and swabbed with povidone-iodine solution. Body heat was maintained both during and immediately after the surgery. Animals were given food and water and were housed individually. All animals were allowed 7-10 days to recover from the surgery and reestablish circadian rhythms.

C. *Diesel Exhaust Generation and Exposure.* DE for exposure experiments was generated using a 4.8 kW (6.4 hp) direct injection single-cylinder 0.320 L displacement Yanmar L70 V diesel generator operated at a constant 3600 rpm. Resistance heating elements provided a constant 3 kW load. Low sulfur diesel fuel (32 ppm), purchased from a local distributor was available from a large storage tank. Engine lubrication oil (Shell Rotella, 15W-40) was changed before each set of exposure tests. From the engine, approximately 85 L/min of the exhaust was directed to a cone diluter and mixed with approximately 595 L/min (7:1 dilution) of high efficiency particulate air (HEPA) filtered room air. The diluted exhaust then traveled approximately 12 m through 7.1 cm diameter stainless steel tubing to a Hazelton 1000 (984 L) exposure chamber housed in an isolated animal exposure room. Target DE concentration of the diluted exhaust was 150 μg of PM/m^3 which was routed to an unfiltered exposure chamber. The chamber concentration was controlled by periodic adjustments of dilution air based on continuous mass concentrations determined by tapered element oscillating microbalance (TEOM, Rupprecht and Patashnick Co., series 1400, Albany, NY) instruments. These instruments include a heated (50 °C) chamber that could theoretically vaporize low temperature volatiles. Control animals were placed in a chamber supplied with the same HEPA filtered room air. The chambers were operated at the same flow rate (424 L/min) which resulted in approximately 25 air exchanges per hour. Integrated 4 h filter samples (14.1 L/min) were collected daily from each chamber and analyzed gravimetrically to determine particle concentrations. Continuous emission monitors (CEMs) were used to measure chamber concentrations of PM by

TEOM, oxygen (O_2, Beckman Corp., model 755, La Habra, CA), carbon monoxide (CO, Thermo Electron Corp., model 48, Franklin, MA), nitrogen oxides (NO and NO_2, Teledyne Technology Co., model 200A4, San Diego, CA), and sulfur dioxide (SO_2, Thermo Electron Corp, model 43c, Franklin, MA). Samples were extracted through fixed stainless steel probes in the exposure chambers. Gas samples were passed through a particulate filter prior to the individual gas analyzers. Dilution of air was adjusted periodically to maintain target PM concentrations as measured by the TEOM. Particle size distributions were characterized during each exposure using an engine exhaust particle sizer (EEEPS, TSI Inc., model 3090, St. Paul, MN). Chamber temperatures, relative humidity, and noise were also monitored, and maintained within acceptable ranges.

Note: All animals were treated humanely and with regard for alleviation of suffering.

II. Figures

A. Supplemental Material - Figure S1

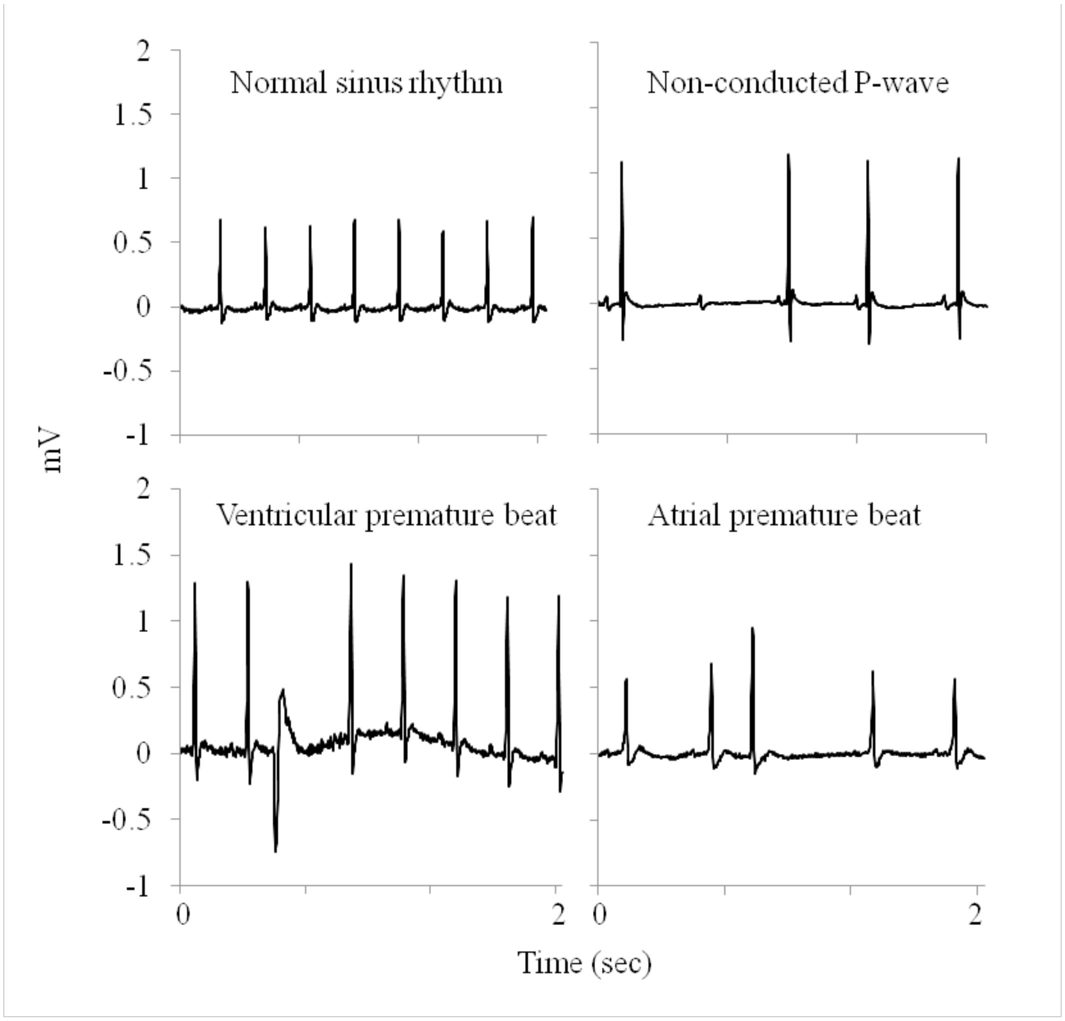

Supplemental Material, Figure S1. Typical electrocardiogram (ECG) traces showing normal sinus rhythm, non-conducted p-wave (NCPW), ventricular premature beat (VPB), and atrial premature beat (APB).

B. Supplemental Material - Figure S2

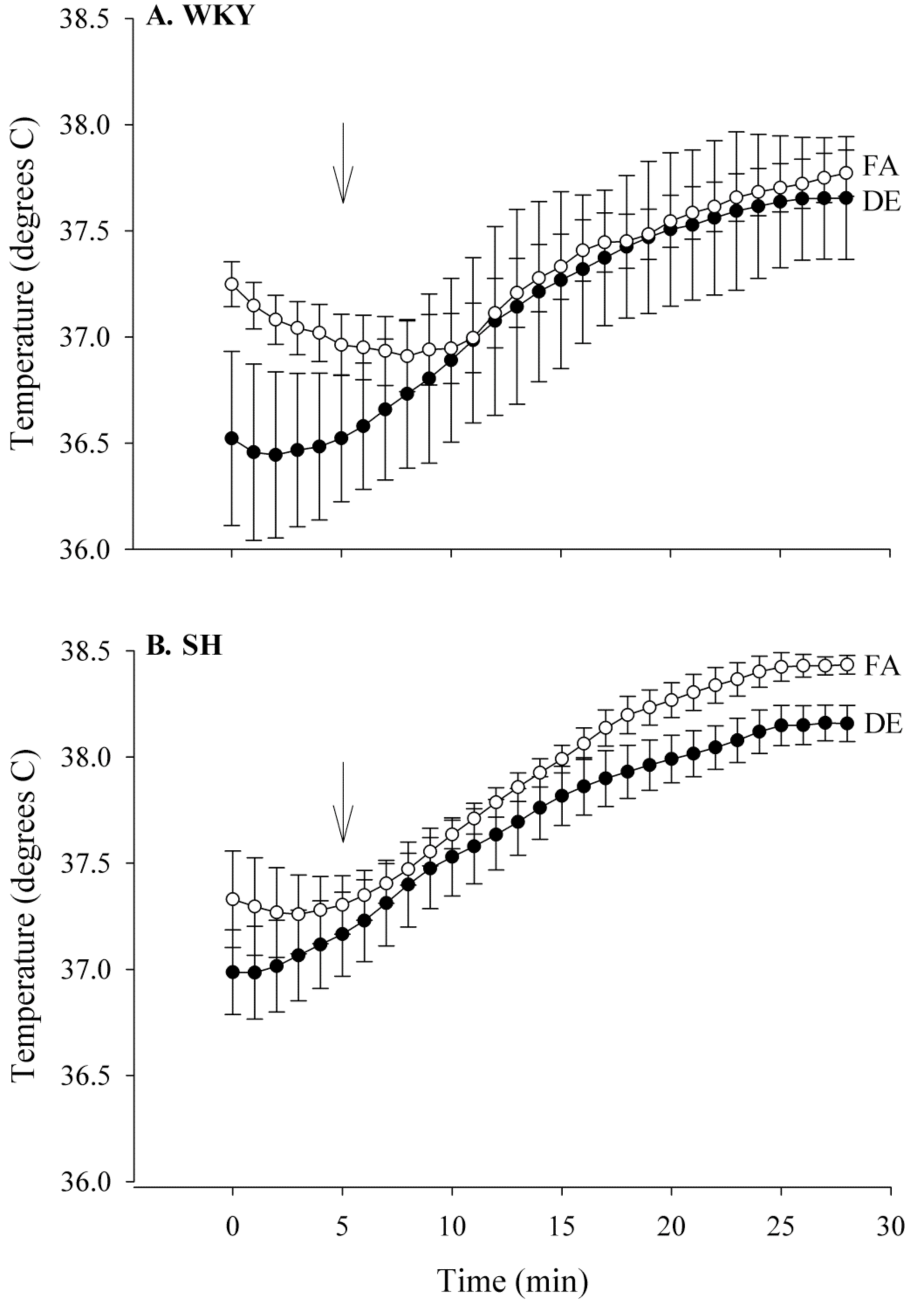

Supplemental Material, Figure S2. Diesel exhaust decreases the baseline temperature of WKY and SH rats. Twenty-four hours after DE, WKY (**A**) and SH (**B**) rats had lower Tco when compared to FA animals. Tco steadily increased in all animals during dobutamine challenge. Arrow = start of dobutamine challenge. Values are mean ± SEM; $p < 0.05$, n = 5-6.

C. Supplemental Material – Figure S3

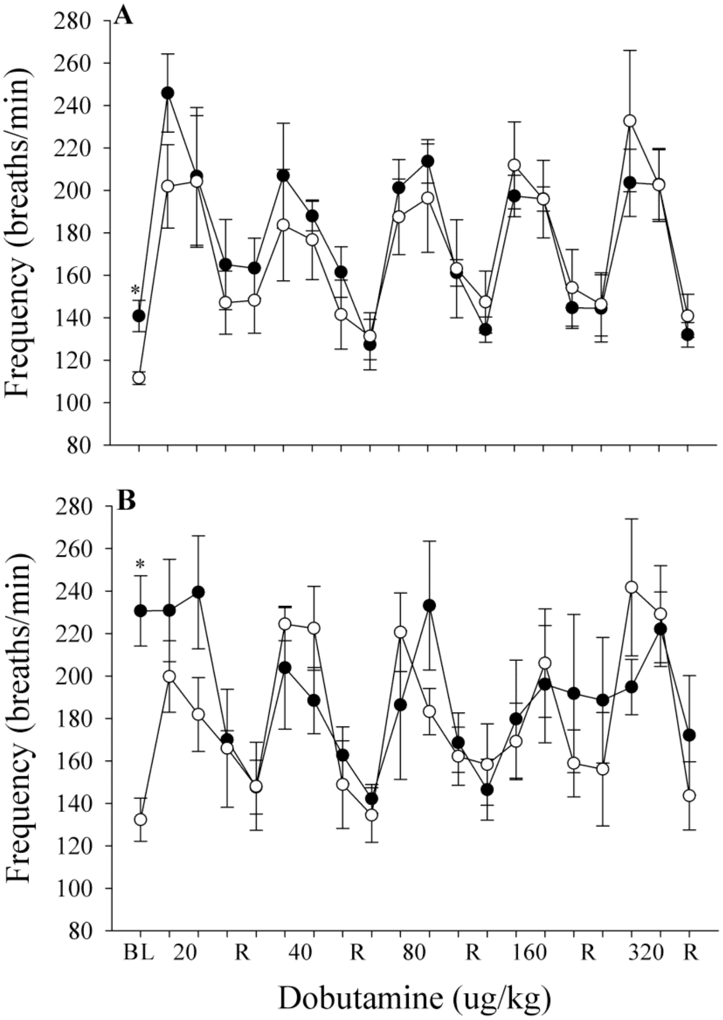

Supplemental Material, Figure S3 - Exposure to diesel exhaust increases the baseline breathing frequency of WKY and SH rats. Dobutamine dose-dependently increased *f* in FA-exposed (unfilled circles) WKY (**A**) and SH (**B**) rats; DE (filled circles) did not appear to affect this response in either strain. However, baseline *f* was significantly increased in WKY and SH rats exposed to DE, and remained slightly elevated for SH rats during post-challenge recovery. Values are mean ± SEM; * significantly different from FA controls; $p < 0.05$, n = 5-6.

D. Supplemental Material - Figure S4

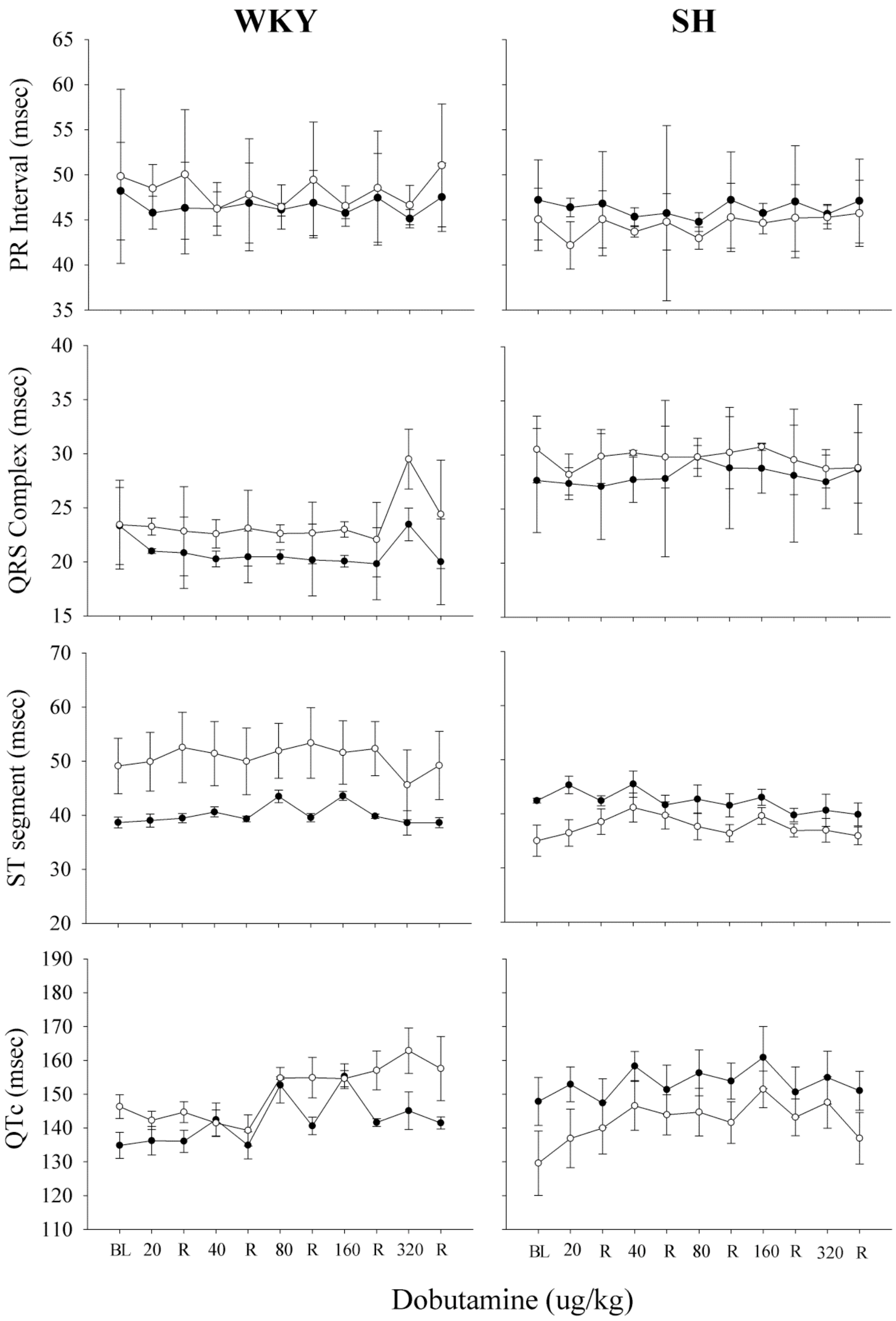

Supplemental Material, Figure S4. Electrocardiogram during dobutamine challenge. There was no difference in the PR interval of WKY and SH rats, whether exposed to FA (unfilled circle) or DE (filled circle). SH rats had higher QRS intervals than WKY rats; exposure to DE did not cause the QRS interval to change significantly in either strain. Exposure to DE caused ST segment and QTc to decrease in WKY rats during dobutamine challenge when compared to FA. DE had no effect on ST segment in SH rats, but did cause QTc prolongation during the challenge. Values are mean ± SEM; $p < 0.05$, n = 5-6.

III. References

Hazari MS, Haykal-Coates N, Winsett DW, Costa DL and Farraj AK. 2009. A single exposure to particulate or gaseous air pollution increases the risk of aconitine-induced cardiac arrhythmia in hypertensive rats. Tox. Sci. 112(2):532-542.

Walker MJ, Curtis MJ, Hearse DJ, Campbell RW, Janse MJ, Yellon DM, et al. 1988. The Lambeth Conventions: guidelines for the study of arrhythmias in ischaemia, infarction, and reperfusion. Cardiovasc. Res. 22:447-455.
